# Viral Dynamics and Real-Time RT-PCR Ct Values Correlation with Disease Severity in COVID-19

**DOI:** 10.3390/diagnostics11061091

**Published:** 2021-06-15

**Authors:** Ali A. Rabaan, Raghavendra Tirupathi, Anupam A Sule, Jehad Aldali, Abbas Al Mutair, Saad Alhumaid, Muzaheed, Nitin Gupta, Thoyaja Koritala, Ramesh Adhikari, Muhammad Bilal, Manish Dhawan, Ruchi Tiwari, Saikat Mitra, Talha Bin Emran, Kuldeep Dhama

**Affiliations:** 1 Molecular Diagnostic Laboratory, Johns Hopkins Aramco Healthcare, Dhahran 31311, Saudi Arabia; arabaan@gmail.com; 2 Department of Medicine Keystone Health, Penn State University School of Medicine, Hershey, PA 16801, USA; drraghutg@gmail.com; 3 Department of Medicine, Wellspan Chambersburg and Waynesboro Hospitals, Chambersburg, PA 17201, USA; 4 Department of Informatics and Outcomes, St Joseph Mercy Oakland, Pontiac, MI 48341, USA; anupam.a.sule@stjoeshealth.org; 5 Pathology Organization, Imam Mohammed Ibn Saud Islamic University, Riyadh 13317, Saudi Arabia; jaaldali@imamu.edu.sa; 6 Research Center, Almoosa Specialist Hospital, Al-Ahsa 36342, Saudi Arabia; abbas4080@hotmail.com; 7 College of Nursing, Princess Norah Bint Abdulrahman University, Riyadh 11564, Saudi Arabia; 8 School of Nursing, Wollongong University, Wollongong, NSW 2522, Australia; 9 Administration of Pharmaceutical Care, Al-Ahsa Health Cluster, Ministry of Health, Al-Ahsa 31982, Saudi Arabia; saad4eternity@yahoo.com; 10 Department of Clinical Laboratory Science, College of Applied Medical Sciences, Imam Abdulrahman Bin Faisal University, Dammam 31441, Saudi Arabia; marasheed@iau.edu.sa; 11 Department of Infectious Diseases, Kasturba Medical College, Manipal Academy of Higher Education, Manipal 576104, India; nityanitingupta@gmail.com; 12 Department of Internal Medicine, Mayo Clinic Health System Mankato, Mayo Clinic College of Medicine and Science, Mankato, MN 56001, USA; koritala.thoyaja@mayo.edu; 13 Department of Hospital Medicine, Franciscan Health Lafayette, Lafayette, IN 47905, USA; ramesh.adhikari@franciscanalliance.org; 14 School of Life Science and Food Engineering, Huaiyin Institute of Technology, Huaian 223003, China; bilaluaf@hotmail.com; 15 Department of Microbiology, Punjab Agricultural University, Ludhiana 141004, India; dhawanmanish501@gmail.com; 16 The Trafford Group of Colleges, Manchester WA14 5PQ, UK; 17 Department of Veterinary Microbiology and Immunology, College of Veterinary Sciences, Uttar Prade Pandit Deen Dayal Upadhyaya Pashu Chikitsa Vigyan Vishwavidyalaya Evam Go Anusandha Sansthan (DUVASU), Mathura 281001, India; ruchi.vet@gmail.com; 18 Department of Pharmacy, Faculty of Pharmacy, University of Dhaka, Dhaka 1000, Bangladesh; saikatmitradu@gmail.com; 19 Department of Pharmacy, BGC Trust University Bangladesh, Chittagong 4381, Bangladesh; 20 Division of Pathology, ICAR-Indian Veterinary Research Institute, Izatnagar, Bareilly 243122, India

**Keywords:** COVID-19, SARS-CoV-2, Ct values, RT-PCR, viral load, severity, mortality

## Abstract

Real-time RT-PCR is considered the gold standard confirmatory test for coronavirus disease 2019 (COVID-19). However, many scientists disagree, and it is essential to understand that several factors and variables can cause a false-negative test. In this context, cycle threshold (Ct) values are being utilized to diagnose or predict SARS-CoV-2 infection. This practice has a significant clinical utility as Ct values can be correlated with the viral load. In addition, Ct values have a strong correlation with multiple haematological and biochemical markers. However, it is essential to consider that Ct values might be affected by pre-analytic, analytic, and post-analytical variables such as collection technique, specimen type, sampling time, viral kinetics, transport and storage conditions, nucleic acid extraction, viral RNA load, primer designing, real-time PCR efficiency, and Ct value determination method. Therefore, understanding the interpretation of Ct values and other influential factors could play a crucial role in interpreting viral load and disease severity. In several clinical studies consisting of small or large sample sizes, several discrepancies exist regarding a significant positive correlation between the Ct value and disease severity in COVID-19. In this context, a revised review of the literature has been conducted to fill the knowledge gaps regarding the correlations between Ct values and severity/fatality rates of patients with COVID-19. Various databases such as PubMed, Science Direct, Medline, Scopus, and Google Scholar were searched up to April 2021 by using keywords including “RT-PCR or viral load”, “SARS-CoV-2 and RT-PCR”, “Ct value and viral load”, “Ct value or COVID-19”. Research articles were extracted and selected independently by the authors and included in the present review based on their relevance to the study. The current narrative review explores the correlation of Ct values with mortality, disease progression, severity, and infectivity. We also discuss the factors that can affect these values, such as collection technique, type of swab, sampling method, etc.

## 1. Introduction

The coronavirus disease 2019 (COVID-19) ongoing pandemic has caused catastrophe in the lives of millions of people worldwide. Several articles since February 2020 report a heterogeneous clinical presentation of this disease [1,2,3,4,5]. There have been rapid and significant advancements in knowledge of the pathogenesis, immunobiology, genomics and molecular biology of the causative virus, i.e., severe acute respiratory syndrome coronavirus 2 (SARS-CoV-2). Additionally, the WHO (World Health Organisation) emphasizes that rapid diagnosis is an essential measurement to combat the devastating consequences of the COVID-19 epidemic. Reliable detection and screening of SARS-CoV-2 are imperative where SARS-CoV-2-positive cases are rising, causing widespread transmission [6]. In this context, to create accurate and reliable diagnostic procedures and a possible vaccination against SARS-CoV-2, many strategies have been adopted [7]. However, certain key areas are still unknown, and validation is required in others. The importance of inflammatory cells in SARS-CoV-2 infection has been recognized. Elevation in neutrophils and inflammatory markers, with a concomitant decrease in lymphocytes, is associated with a poor prognosis [8,9]. Most of the published data focus on variation between severe and non-severe COVID-19 disease [10,11]. There is a need for deeper apprehension of disease pathogenesis theories, which will facilitate in the development of diagnostic tools for risk-stratification and offer potential therapeutic targets in the pathways triggering a hyper-inflammatory cascade [8,9,10,11].

There is conflicting evidence regarding the relation between antibody titers and COVID-19 severity [12,13]. Therefore, more research is needed regarding antibody kinetics to understand the role of antibody-mediated enhancement in COVID-19 pathogenesis [14]. This can also serve as a guide to identifying patients who may benefit from convalescent plasma [15].

SARS-CoV-2 can be isolated from the nasopharynx for 14–21 median days after onset of symptomatic disease [15,16]. This tropism of SARS-CoV-2 for the lower respiratory tract can be due to the abundance of angiotensin-converting enzyme 2 (ACE2) receptors (that serve as viral anchoring points) [17,18]. Reports state that the peak SARS-CoV-2 load is seen during the first seven days of viral infection [19,20]. In several findings, the immunosuppressed personal’s respiratory samples for virus culture can be performed during the first seven days of symptom onset as the viral loads are at their peak, or virus can be cultured from an infected person’s respiratory tract in the first week after symptoms’ appearance [21,22,23]. Transmission of SARS-CoV-2 likely subsides after seven days [24]. However, this must be confirmed in studies with large sample sizes, due to its vital role in isolation guidelines and infection control protocols [25].

Many published papers have suggested that the SARS-CoV-2 viral load can predict the likelihood of disease spread and severity. In the 2002 SARS-CoV epidemic, a higher viral load was related to increased emergency care requirements, intensive care, and overall poor prognosis [26,27]. Several differences have been noted in the recent SARS-CoV-2 pandemic and the SARS-CoV epidemic of the year 2002.

**Figure 1:**
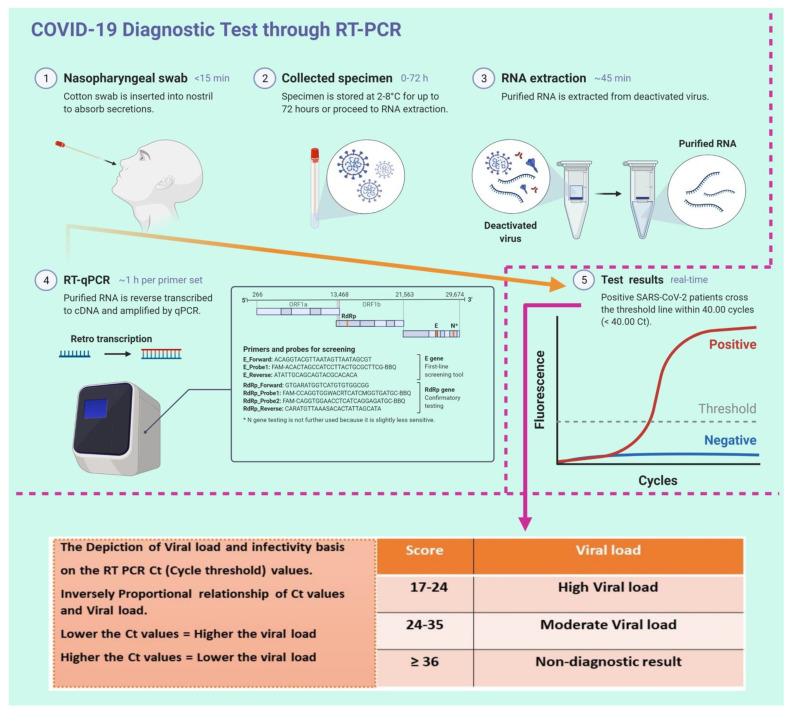
Schematic illustration of RT-PCR methodology and the detection of a positive sample in clinical practice, using a specific threshold and Ct value. The SARS-CoV-2 viral load is inversely proportional to the Ct values, and a lower Ct value corresponds to a high viral load, indicating a higher level of infectiousness. The figure was designed by Biorender.com program (https://biorender.com/, accessed on 15 April 2021).

The SARS-CoV-2 real-time RT-PCR test reports as negative or positive in clinical practice, using a specific threshold for Ct values [28] (Figure 1). This cut-off is calculated by an algorithm that automatically interprets various parameters of the amplification process [26]. 

However, there are some ambiguities, such as whether it is best to use Ct values in predicting the level of infectivity and disease severity in patients infected with SARS-CoV-2. In addition, one class of scientists has questioned whether RT-PCR be termed a gold standard technique for the detection of SARS-CoV-2 infection [29,30]. Furthermore, recent evidence suggests that correct predictions, combined with efficient analysis of Ct values, can pave the way for improved diagnostic methodology, which can then be used to manage and control virus spread as well as monitor disease progression [31,32]. Given that rapid and accurate detection of SARS-CoV-2 is crucial in controlling the outbreak in the community and in hospitals, this review focuses on the concept of Ct values in COVID-19, and its relationship with disease severity, disease progression and infectivity. We also highlight factors affecting these values and present Ct values’ relationship with haematological and inflammatory biomarkers.

## 2. Understanding Ct Values

When the fluorescent signal exceeds the background fluorescence, the thermal cycles are defined as the cycle threshold [33]. This is a semi-quantitative measure that helps in the broad categorization of viral genetic material in patient samples following testing by RT PCR. It can be variable and classified as low, medium, or high. A standard RT-PCR assay runs a maximum of 40 thermal cycles [34]. A low Ct designates an elevated concentration of genetic material, typically correlated with high infection risk [28]. A high value of Ct specifies a lower infectivity risk as it depicts a low concentration of viral genetic material [35]. However, low viral load can also be due to an incubation period or convalescent stage, or primary replication of the virus at other sites in the body (lower respiratory tract, LRT) [28]. Nevertheless, high Ct values with upper respiratory tract (URT) specimens also had suggestive pulmonary computed tomography (CT) scan findings. An A3-point increase in Ct values indicates a 10-fold reduction in viral genetic material quantity [36]. 

## 3. Importance of Determination of Ct Values

Progress in understanding COVID-19 pathogenesis is continuously evolving. Limited data can establish the relation between the patient’s viral loads and prognoses, such as disease progression or death rate. Huang et al. reported an inverse correlation between SARS-CoV-2 Ct value and mortality [37]. During the previous pandemics of the MERS-CoV virus and SARS, a similar association of low Ct values with the severity of infection was reported [25,38]. COVID-19 patients have variable clinical presentations and course of illness. It has been challenging to identify patients who are more likely to have a poorer outcome [26]. RT-PCR-based measurements and the accurate observations of Ct values have been proposed as an effective supplement technique to the chest CT, which is commonly used in the diagnosis of COVID-19 [15]. Ct values have been linked to tissue and organ damage in a recent post-mortem analysis of COVID-19 deceased. However, the magnitude of organ damage was not significantly associated with Ct values. RT-PCR and Ct values have been implicated in obtaining a more comprehensive image of viral dynamics in COVID-19 deceased. Higher Ct values represent a low viral RNA load and a lower risk of infection transmission [39]. On the other hand, a recent study showed that Ct values via successful virus isolation in cell cultures indicated that patients with Ct values beyond 33–34 seem to be no longer infectious [40]. In comparison, it is often proposed that Ct values do not correlate strongly with infectivity [39]. In another study, the epidemiologic correlations of Ct values with COVID-19 were studied. Ct values have the ability to be a basic and readily available method for predicting and modelling epidemiologic dynamics at the population level. More demographic research and predictive models based on international datasets are needed [41]. All these findings suggest that there may be several benefits associated with the indications provided by Ct values, as they may provide insights into the prognosis and consequences of the infection.

## 4. Scenarios Corresponding to High Ct Values

High Ct values are found in:

◆ Asymptomatic infection with unknown infectivity risk where the stage of the disease is not known [8].
◆ Pre-symptomatic infection that subsequently may develop into symptomatic infection with high viral load and infectivity [42].
◆ During acute COVID-19 with a high risk of infectivity, if the samples collected are insufficient or degraded samples (sub-optimal sample storage and handling techniques) [43,44].
◆ Symptomatic persons should be assumed to be potentially infectious within the first 10 days of onset of disease, and asymptomatic persons for 10 days after the positive swab result [45].
◆ During the convalescent phase of COVID-19 when the viral load is diminished. Persistence in detecting viral genetic material (that is likely non-infectious) has been observed for SARS-CoV-2 [46].
◆ Immunocompromised and hospitalized individuals with a critical illness are more likely to shed potentially infectious viruses for longer [18,47].

While high Ct values may be associated with a low viral load and reduced infectivity, a swab taken at a single point in time does not provide details about the trajectory or subsequent illness course [27]. Some asymptomatic cases of SARS-CoV-2 RT-PCR have reported Ct values comparable to symptomatic patients [48]. Another study reported an earlier reduction in Ct values in asymptomatic cases rather than symptomatic cases [49]. Ct values in some patients were like those in pre-symptomatic and symptomatic phases [50,51]. 

## 5. Factors Affecting Ct Values

Firstly, recent evidence has shown that in some cases, diagnostic tests might fail to detect positive cases, especially 3–4 days after infection, and after 10–12 days of infection, alerting us to the fact that any asymptomatic individual with negative test results can be infectious [52]. In addition, Ct values are commonly affected by pre-analytic, analytic, and post-analytical variables. The pre-analytic variables include collection technique, specimen type, time of sample taken and viral kinetics, the difference between the viral load in URT and LRT samples, transport and storage conditions before the testing, and the specimen age [53,54]. The analytic variables include extraction efficacy of nucleic acid, viral RNA load in the collected samples, primer design, nature of the target RNA, real-time PCR efficiency, and Ct value determination method [55]. Post-analytical variables include interpretation and reporting of the results [55]. Aside from these considerations, thermal inactivation has been identified as a critical factor influencing the RT-PCR outcome. In this context, a recent study reported increased Ct values in RT-PCR tests from COVID-19 patients after thermal incubation. Approximately 50% of the weak-positive samples gave negative results instead of positive results after heat inactivation. Hence, it was established that thermal inactivation reduced RT-PCR performance for viral detection by a significant level [56]. Considering these factors, standardization of Ct values becomes essential for appropriate interpretation [57]. Some of these factors are explained as follows:

### 5.1. Pre-Analytic Variables Affecting Ct Values

#### 5.1.1. Collection Technique

The collection technique plays a vital role in correct Ct values. The patient should be adequately prepared for the best specimen collection. The patient should avoid blowing the nose or holding nasal medications upon awakening when the specimen is collected from the nasopharynx. If the sample is taken from the throat or the internal part of the cheek, the patient should not eat or drink anything after waking up, and primarily, should not brush teeth or gargle. Dacron or polyester flocked swabs should be used for the sample collection [53]. The Ost-collection samples must reach the laboratory in a timely fashion [49]. A faulty collection method breaching aseptic precautions may lead to contamination of the sample. Ct ≥ 36 should be treated as a non-diagnostic result, which may be caused by poor material collection, sample contamination at the RNA isolation stage, too early or too late material collection during the disease course, etc. To avoid errors in interpretation, each sample should be analyzed in triplicate. Hence, the European Centre for Disease Prevention and Control (ECDC) has recommended that there is a need for retesting of samples having Ct > 36 to rule out the possibility of contamination [54].

#### 5.1.2. Type of Specimen

The Ct values and load of SARS-CoV-2 detection in swabs are affected by the collection site [49,58,59]. Nasopharyngeal swabs are the most specific and accurate swab site for COVID-19 diagnosis, followed by throat swabs [60]. Higher viral loads (inversely proportional to Ct values) were detected in nasal samples compared to throat specimens [8]. Yang et al. reported that the viral RNAs in bronchoalveolar lavage (BAL) from severe COVID-19 cases were beneficial for diagnosing and monitoring these cases [61]. Sputum and BAL have shown higher sensitivity in comparison to upper respiratory samples, probably due to the presence of greater viral loads in these specimens [62]. However, a suction tool and an expert operator are needed to collect BAL [49]. The procedure is also invasive and painful to the patients [49]. Unlike BAL samples that require expertise, other routine laboratory samples such as nasal swab, throat swab, and sputum are convenient and safe to collect [61].

In a research study, salivary samples of two patients detected positive, whereas respiratory swabs collected on the same day had negative results [63,64]. This is a concern in the isolation of patients post-discharge from the hospital. Some could be contagious and could spread the infection through saliva even though their nasopharyngeal samples were negative for two consecutive days [65]. Therefore, SARS-CoV-2 may be transmitted through saliva directly or indirectly, even among patients without coughing or other respiratory symptoms [66]. Furthermore, the lower the Ct values in salivary RT-PCR, the higher the patient’s LDH levels recorded on the same day. Thus, the salivary viral load is directly proportional to serum LDH [67]. Therefore, saliva RT-PCR may serve a diagnostic and a prognostic purpose [63]. Woffel et al. showed that cases had a difference of > 3 Ct value between the swab samples and the sputum samples [68]. A higher viral load in swab samples was seen in two cases, and in two cases the sputum had a higher viral load. The remaining three cases were equivocal [68].

Hence, two or more distinct sampling sites may increase the chances of viral detection.

#### 5.1.3. Sampling Time and Viral Kinetics

In two patients, viral load kinetics of SARS-CoV-2 infection were evaluated and found to be different from earlier published coronavirus studies [63]. With progression in disease severity from mild to severe, viral load in the URT sample can change. A decrease in the URT and synchronous increase in the LRT reflect an anatomical transition and disease severity [68]. It was observed by Liu et al. that URT samples were more sensitive for virus detection in the initial stages of the disease in febrile COVID-19 cases compared to later stages [69]. As LRT samples are difficult to obtain in early disease, throat wash gargles could be used as an alternative [70].

Tahamtan et al. detected the virus from a patient’s URT on day two and LRT on day three of symptom onset [49]. There was an increased viral load on the fifth day in LRT samples. On the seventh day, the viral load decreased in both URT and LRT samples. On 13th and 14th days, the RT-PCR was positive (low viral load) in LRT and URT samples. The viral load was undetectable for the next two consecutive days for both samples. SARS-CoV-2 was detected in both LRT and URT samples two weeks after the onset of the symptom. However, they noted that the second patient’s viral load was lower than in the first patient. On the 18th and 20th day, the samples were converted to negative URT and LRT, respectively. However, on day 25, the URT specimen for genes E and RdRp was positive. This variation between the two cases demonstrates the variation in viral load kinetics, indicating that the time of sample collection and progression of the disease course play a crucial role in RT-PCR results. 

### 5.2. Analytic Variables Affecting Ct Values

#### 5.2.1. Internal Control

For an RT-PCR sample to be negative, there should be no fluorescence growth curves crossing the threshold line in the negative template control (NTC) sample [49]. However, there can be false-positive results, with some primers suggesting contamination of the sample [49]. The selection of the correct reference gene to normalize the results is a critical step in the RT-PCR method. Inaccurate data is likely to be generated if the reference gene is not correctly selected [49]. Identifying contaminants that may interfere with the nucleic acid extraction and amplification process is crucial and can be addressed with strict internal control measures [49]. This can be achieved by following standardized guidelines for reporting and confirmatory testing of RT-PCR [49,71]. 

Consequently, raw Ct data as inaccurate references are being utilized to interpret the results. As a result, using raw Ct values along with inaccurate internal control to predict SARS-CoV-2 viral load and associate it with various outcomes might lead to incorrect results [49]. In this context, the following controls should also be run in parallel for each RT-PCR plate: (1) negative control to assess the purity of the reagents, (2) positive control to check if false negatives are being detected in the experiment, and (3) internal control to indicate perfect nucleic acid extraction and quality of samples and PCR.

#### 5.2.2. Type of RT-PCR

Qualitative RT-PCR is generally applied to identify the SARS-CoV-2 virus, whereas quantitative RT-PCR quantifies the viral load [72]. Due to the huge diversity and inconsistency of the standard curves and viral load estimation from samples with serial dilution, a two-step approach is recommended, where both qualitative and quantitative tests are done [73]. This approach will improve the test’s accuracy, as a standard curve with a suitable detection limit for viral quantification will appropriately follow the titers and viral kinetics [64,73,74]. Test results have important implications as false-negative results may cause a risk of transmission of infected people and for the management of COVID-19 [75].

#### 5.2.3. Purity of Reagents

The reagents used for RT-PCR can be contaminated. This can give rise to errors in the detection of the virus. Therefore, it is essential to have regular quality checks to ensure the purity of the reagents [74].

#### 5.2.4. Pipetting Defects

Due to human errors or defects in an automated machine, pipetting errors can give rise to false-positive RT-PCR results. These errors can be controlled with positive and negative controls on each run of the assay and with a review of the controls before determining the results [73].

### 5.3. Post-Analytical Variables

#### Interpreting the Reports

Reporting and interpreting the results plays a pivotal role in detecting viral load in RT-PCR. False-positive results can be due to errors in interpretation of amplification curves [76] or errors in transcription [77]. These errors could be avoided by reviewing and re-checking the results and interpretation before reporting.

## 6. Impact of SARS-CoV-2 Variants on Diagnosis

Due to sequencing efforts, monitoring, and surveillance of variation within the novel coronavirus genome, many variants of concern (VOCs) have been identified, that have affected virus biology and disease transmission. Across all virus genomes sequenced to date, thousands of mutations have emerged since the start of the pandemic, resulting in the rise of a range of distinct variants. Most of these variants do not affect the virus biology, yet, more recently, several variants have been identified that appear to increase transmissibility and potentially have an impact on disease severity, and these are called variants of concern VOCs [78]. When mutations occur at primer binding sites or affect the structure of viral antigen targets that are detected by the antigen tests, they have the potential to impact the accuracy of diagnostic tests [79]. In addition, genetic variability and recurrent mutations possess a severe risk of loss of sensitivity of different published assays for the detection of SARS-CoV-2 [80]. For example, for the B.1.1.7 variant, the mutation blocks, in certain PCR tests, the binding between the primers and S-gene targets, resulting in a negative result for this target or S-gene target failure/drop-out [81]. However, most tests use multiple genetic targets, not affecting the accuracy of the test. The mutations in the other genes of the variants do not appear to have impacted diagnostic tests [82]. Therefore, it is better to assess the effect of mutations in all genomic regions, where the majority of tests, for example, do not use the S-gene as a primary target [83]. These variants are only detected via sequencing of the samples after positive RT-PCR. More recently, newly designed multiplexing probe-based qPCRs have allowed the detection of up to four different target DNA to distinguish the current variant in a single PCR reaction, less time consuming when compared to sequencing [84]. Furthermore, Korber et al. proved that the G614 variant’s rapid spread and persistence was associated with higher infectivity in addition to higher viral loads, but not with increased disease severity. Besides, G614 had a lower Ct required for detection [85].

## 7. Correlation of CT Values with Disease

### 7.1. Mortality

Individuals with higher SARS-CoV-2 viral load demonstrated lower Ct levels, correlated with high mortality in patients with COVID-19 (*n* = 875) in Brazil [86]. Magleby et al. unveiled that higher viral load was related to increased intubation risk and higher mortality rates. The authors reported a significant difference between Ct values in COVID-19. 35% patients had a high viral load with Ct lower than 25, 18% had a medium viral load with Ct between 25–30, and 6% had a low viral load with Ct higher than 30 [77]. In a study conducted on hospitalized COVID-19 cases in China, deceased patients had lower average Ct values through the disease course than recovered or hospitalised patients (34.79 versus 37 and 36.97, respectively; *p* < 0.001) [38]. Fajnzylber et al. reported an association between the plasma markers of inflammation and viral load from the respiratory tract. Viral load could be considered a predictor of morbidity and mortality [76]. Despite a continuous rise in clinical information related to COVID-19, very few studies elaborate on viral load’s correlation with disease progression or mortality [28]. Association between SARS-Cov-2 Ct value and mortality has been reported in only one study and demonstrated the correlation of lower Ct values with high mortality risk [37]. These findings are in agreement with data from earlier epidemic-inducing CVs [27,38]. Therefore, it is imperative to keep assessing the SARS-CoV-2 Ct value with the availability of further data. Eleven reports found a correlation between Ct values and symptoms severity. Among these, seven studies indicated lower Ct values with disease severity, which agrees with earlier studies related to Ct values in different respiratory infections [16,38,87]. A significant study involving 5830 COVID-19 patients determined that viral load through Ct values in nasal swabs of symptomatic and asymptomatic patients was statistically insignificant [88]. 

Relatively low Ct values upon detection (i.e., considerably higher viral load) were related to considerably increased fatality rates among both in- and out-patients, according to recent observational research done between March and May 2020 at a large quaternary academic medical center in New York City, USA. It is worth noting that individuals with a shorter period between symptom start and testing had poorer prognoses. Those who presented less than three days after symptom start having a two-fold greater risk of mortality. Ct levels remained a robust predictor of fatality rates among patients from the initial diagnosis of SARS-CoV-2 infection [89].

### 7.2. Disease Severity

In mild and severe COVID-19 cases, analysis of respiratory samples detected a minimum level of almost 10 copies of vRNA/μL [2]. Júnior et al. (*n* = 43) reported an average Ct value of 34.92 from asymptomatic individuals in Brazil [90].

A Chinese study found that severe cases have higher viral load and longer virus persistence [17,91]. In severe and critical diseases, the viral load remained consistently high over the disease course, with persistent low Ct values [92]. Although some studies have reported an association between disease severity and Ct values [13,17,93], another study reported no difference in mean viral loads among patients with and without pneumonia [94]. Pneumonia patients had high viral load only in the female subgroup, and the elevated CRP and serum amyloid A positive subgroups [94].

To study the association between virus persistence and disease severity, the method of detecting viral RNA in non-survivors until death was used [94,95,96,97]. RNA detected via RT-PCR does not provide information on its infectious capacity. Furthermore, no live virus was cultured from a specimen obtained eight days post-symptoms onset [68,72]. In other words, viral RNA’s persistence indicates the immune response status (RNA clearance) and not the disease severity [98]. Recent research has revealed that there could be a relationship between Ct values and disease severity or associated morbidity with COVID 19, as patients who died had substantially lower Ct values compared to patients who recovered, but these patients had a considerably shorter period with symptoms before testing [99]. The sample size of the study was too small to show a strong positive or negative association between Ct value and disease severity and fatality rate using statistical analysis.

On the other hand, a retrospective cohort analysis in New York by Zacharioudakis et al. (*n* = 42) showed that a three-fold increase in Ct value denoted a correlation with a 0.15 improvement in terms of genomic load on Sequential Organ Failure Assessment (SOFA) score [100,101,102,103,104,105,106]. Another larger study with 192 patients revealed that patients who died in hospital had significantly lower Ct values than those who were discharged alive [100]. The same was true for patients who needed admission to the ICU or developed shock, having significantly lower Ct values than those who did not. Ct values had a statistically significant negative correlation with the length of ICU stay.

### 7.3. Co-Morbidities

Poor prognosis of COVID-19 case management is reported due to various co-morbidities. Prolonged viral shedding is seen in mild COVID-19 patients with older age, hypertension, and diabetes mellitus [13,101].

Advanced age has been directly correlated with increased viral load. Immune-senescence and immunocompromised status are hindrances to mounting a full immune response, leading to a longer asymptomatic incubation period [102]. In hospitalized patients with worse prognosis (as predicted by older age high SOFA score and d-dimer > 1 kg/mL), there was an increase in viral load, and Ct values were low up to the third week of infection. Afterwards, a decrease in viral load and negative RT-PCR results were observed in the third to sixth week [103,104]. Researchers also observed a substantial relationship between elevated virus load and smoking status and recent chemotherapy [91]. A great deal of evidence has correlated early Ct values from a nasopharyngeal swab with disease susceptibility (age and various comorbidities, including hypertension and smoking [105,106] and predicted sequelae, survival, and disease severity in symptomatic patients [107]. Recently, Kostakoglu et al. [102] assessed the diagnostic value of CT and rtRT-PCR in COVID-19 patients. Initially, the rtRT-PCR test was detected positive and negative in 142 (Group-I) and 61 patients (Group-II), respectively. Notably, rtRT-PCR positivity was associated negatively with CT values, age, and comorbidity, breathing shortness and duration of symptoms, whereas it was positively linked with headaches. On the other hand, CT values were positively related to fever, comorbidity, age, and breath shortness.

### 7.4. Biochemical and Haematological Markers

An association between Ct value and haematological and biochemical markers has been reported [38,94,108,109,110]. Some studies have reported an association between lower Ct values and higher lactate dehydrogenase levels [38,40,111]. Severe COVID-19 cases having lower Ct scores had low lymphocyte counts and T-cell counts but significantly increased total and differential neutrophil counts [40,112]. On the other hand, Yuan et al. negatively correlated Ct with neutrophil counts [111]. 

Patients with lower Ct values had lower serum albumin concentrations, higher creatinine kinase myocardial, and high-sensitivity troponin 1 levels [40,61]. Liu et al. established a negative correlation between CRP and Ct value with an r = −0.584 [112], but Yuan found no significant correlation [111]. Other biochemical markers (such as calcium, inorganic phosphorus, N-terminal pro-brain natriuretic peptide) and haematological markers (such as myoglobin, 14 IL-2R, angiotensin II, eosinophil/basophil counts) were also associated with Ct values [38,40,111].

It must be noted that Fajnzylber et al. reported that, in 88 hospitalized patients with COVID-19, increased detection of plasma viral load was significantly related to a more severe respiratory disease course, lower lymphocyte counts, and high inflammation markers [92].

### 7.5. Infectivity and Duration of Symptoms

Researchers from France have documented the advantages of Ct values in determining the infectivity of COVID-19 [40]. Salvatore et al. (*n* = 93) demonstrated lower values in patients presenting with respiratory symptoms [112]. Similar findings were reported when live viral loads were isolated from patients with COVID-19 eight days after symptom onset [21]. In an investigation by Singanayagam et al. (*n* = 324), CT values were greatest during the symptomatic phase, followed by a steady decrease in the first 10 days post-infection, with a subsequent plateauing [111]. These patients had low Ct levels within the infection period (lower in week 1 than week 2) [113]. Yu et al. reported a negative correlation between the disease prognosis and the Ct values in mild to moderately ill patients with COVID-19 admitted in hospitals [114].

Various respiratory sample analyses reported the highest values of viral loads during the initial week of symptoms [113,115,116,117,118] or at day 3 to 5 of illness onset [119] followed by a continuous decline.

Zou reported an RT-PCR analysis for *N* and *Orf 1b* genes in the COVID-19 case, showing Ct values of 30–32 on days 7, 10, and 11 post-contact [8]. La Scola et al. noted that, despite the possibility of virus detection using PCR after 20 days of symptoms, viral isolation in culture was not feasible after day eight. This was true even with high viral loads [117]. Arons et al. concluded that the virus is viable from 6 days pre-symptom onset to 9 days after symptom onset, in the presence of high viral load in asymptomatic cases, of which 50% were verified COVID-19 positive [120].

RT-PCR findings may remain positive for weeks, despite the resolution of the symptoms [121]. 34% of patients remained RT-PCR positive even after 40 days [104].

Persistent positivity on PCR and its lack of ability to distinguish infectivity is a problem plaguing many public health authorities when making recommendations for isolation and quarantine. A study by Bullard et al. (*n* = 90), found a significant association between a lower Ct value and the capability of recovering from infectious virus, with an OR value of 0.67 (95% CI = 0.58–0.77) [105]. Singanayagam A et al. (*n* = 324) assessed that the likelihood of recovery from the virus with a Ct higher than 35 was only 8% (95% CI = 2.8%–18%) [96]. Currently, a time-based approach is used in many facilities to discontinue isolation [100].

Mowrer et al. 2021 showed that a combination of symptom resolution with cycle threshold values might shorten the need for isolation in many patients, improving the stewardship of scare resources [122]. Thus, using Ct values rather than positive/negative reports may offer a more prudent alternative to discontinuation of isolation decisions.

At present most airlines require a negative test within 72 h of the start of travel [123]. A double test screening protocol (one test prior to the start of travel and a second test 5 to 7 days after arrival and sequestration in a quarantine facility) has the potential to reduce cross-border transmission of variants. Suppose cycle thresholds are integrated into this double screen protocol, then such cases will prevent those with a prior history of COVID from being unduly burdened by false positive results [124].

### 7.6. Variants

Variants pose a challenge in controlling this pandemic [125]. Mutations in the viral spike glycoprotein increase the ability to bind to ACE-2 receptors leading to increased disease transmission and severity. As Coronavirus mutates rapidly, all lab manufacturers need to ensure their primers are not affected by the mutations. A mutation in the primer binding segment may lead to the inability of a PCR test to detect that variant. A high vigilance, with surveillance for mutants and frequent genotyping of the virus, particularly in countries experiencing surges, is necessary to preserve the validity and utility of PCR as the gold standard test for detecting COVID. The use of multiple primers may help address the issue of a false-positive result due to another coronavirus that may share that particular genomic sequence [126], as well as false negatives due to a variant with a mutation in the primer binding region. Recently, multiple assays in a qRT-PCR have been recommended to reduce the probability of losing sensitivity of the qRT-PCR test due to the presence of variants. It has been found that single mutations influencing annealing in any PCR test were found in 11,627 (34.38%) of genomes. In 8773 genomes, 25% high-risk variations were found. In contrast, low-frequency single mutations were found in 8% of genomes and were projected not to affect sensitivity. More than 99% of genomes matched with 100% coverage of all oligonucleotides, and significant variants were examined with no loss of sensitivity. As a result, it has been concluded that modifications such as multiple assays can be implicated and amended with Ct values to reduce the potential of sensitivity loss owing to the unknown mutation rate during the current SARS-CoV-2 outbreak [80].

The number of variations of the SARS-CoV-2 virus will continue to rise. Although instances of enhanced infectivity have emerged, there has been no effect on COVID-19 pathogenicity so far. Previous studies have indicated that the multi-target qRT-PCR test for SARS-CoV-2 detection could reduce the risk of sensitivity loss. In this context, assay makers must maintain constant monitoring of genomic variations to respond quickly, if the need for test redesign develops [127]. Further, the redesigned methods must be understood in the context of Ct values for more insightful results. 

### 7.7. Variants and Testing

The presence of SARS-CoV-2 genetic variants can potentially change the performance of the SARS-CoV-2 test. Molecular tests designed to detect multiple SARS-CoV-2 genetic targets are less susceptible to the effects of genetic variation than tests designed to detect a single genetic target. One consideration of how the mutations in these strains will impact molecular test performance depends on whether the test detects nucleocapsid (N) or spike (S) genes. The test targeting S gene may have more chances of false negatives. Major variants, including B.1.1.7, P1, and B1.351, are being detected by most tests as per manufacturers’ reports (https://www.path.org/programs/diagnostics/covid-dashboard-implications-variants-covid-19-molecular-test-detection/ (accessed on 20 May 2021)). Less is known about the B.1.617, the variant recently first identified in India.

Negative tests should be interpreted with caution in a patient with history and physical findings consistent with COVID-19. A repeat test from a different platform and with different genetic targets is recommended in that scenario. Periodic in-silico assessment (computer simulations) of molecular tests by regulatory authorities is recommended to assess the potential impact of genetic variation on the performance of the commercially available testing. Manufacturers and regulators will need to remain vigilant to ensure they keep up with a constantly changing virus. If variants begin to evade detection, it may have far-reaching consequences, not only for that individual patient but for broader public health.

Furthermore, several variables in specimen collection and processing may influence the Ct value outcome. Besides, the magnitude of the disease was determined by the host immune response, regardless of the number of viruses present. There was no statistically meaningful difference between the Ct values of moderately and extremely ill patients with COVID-19, so the results cannot be considered very significantly [92]. However, future studies with large sample sizes may decipher the exact relationships of Ct value with viral load and disease severity.

### 7.8. Recovery

A study by Bullard et al. (*n* = 90), found a significant association between a lower Ct value and the capability of recovering from infection, with an OR value of 0.67 (95% CI = 0.58–0.77) [128]. Singanayagam et al. (*n* = 324) assessed that the likelihood of recovery of the virus with a Ct higher than 35 was only 8% (95% CI = 2.8–18%) [128,129]. A retrospective cohort analysis in New York by Zacharioudakis et al. (*n* = 42) showed that a three-fold increase in Ct value denoted a correlation with a 0.15 improvement in terms of genomic load on Sequential Organ Failure Assessment (SOFA) score [129].

## 8. Conclusions and Future Perspectives

The ideal diagnosis for SARS-CoV-2 infection or COVID19 depends on several factors, such as selecting appropriate and widely available tools and techniques, the most suitable sample form, appropriate sampling techniques, and duration of the infection. In this context, the RT-PCR technique has been exploited widely, and Ct values are considered as the indicators of viral load and may have a role in predicting the disease’s prognosis and severity. The sampling technique, sample time, sample site, disease stage at which sample is obtained, and duration from sampling to analysis play a vital role in Ct values. Ct values have a strong association with multiple biomarkers. Lower Ct have been correlated with higher inflammatory biomarkers, lower lymphocyte and T-cell counts, lower serum albumin levels, higher creatinine kinase, and high-sensitivity troponin 1 levels. Ct values were related to disease severity, mortality, infectivity, and even recovery. Exploring current literature data revealed that Ct values might offer healthcare professionals and clinicians potential advantages in making appropriate decisions for COVID-19 patients. In addition, it is also helpful as a guide to controlling COVID-19 infections and making decisions regarding occupational and public health. Despite the availability of large datasets supporting the importance of Ct values in predicting disease severity and viral dynamics, it must be recognized that there is an urgent need to provide significant evidence supporting the fact that Ct values in all patients with COVID-19 infection can help in better understanding the disease’s dynamics. Moreover, some clinicians and researchers have recommended using Ct values in predicting viral load, which can be correlated with disease severity and the infectivity potential of the subject found positive for SARS-CoV-2 infection.

In addition, calculating viral load is critical for developing antiviral therapy, immunization, and epidemiological control methods for COVID-19. Furthermore, identifying individuals with high viral loads can help doctors better understand risk variables, including age, comorbidities, the severity of symptoms, and hypoxia, which can help them decide whether or not patients need to be sent to the hospital. Several researchers are now examining viral load in various types of samples and investigating its link with clinical outcomes and viral transmission routes. However, cycle threshold (Ct) values alone are frequently utilized as viral load markers in a growing amount of research, which may not be a trustworthy strategy. Normalization of Ct values affects the interpretation of SARS-CoV-2 viral load from various biological samples. Furthermore, many modifications, such as multiple assays, should be considered to prevent the loss of sensitivity of the RT-PCR test due to the emergence of various mutants of SARS-CoV-2.

In the future, there is an utmost need to develop reliable and efficient methods to estimate viral load, and further studies depicting the relationship between viral loads with viable viruses and disease severity/infectivity are also needed. It should also be considered that decisions about predicting the severity of disease, fatality rate, and viral dynamics should be based on a wide range of clinical parameters such as age, co-morbidities, and biomarkers, namely lymphocyte counts, C-reactive protein level, and D-dimer level, as there are several question marks and knowledge gaps associated with a positive correlation between Ct values and disease severity, which is yet to be explored.

## Data Availability

Available data are presented in the manuscript.
